# The value of multiparameter ^18^F-FDG PET/CT imaging in differentiating retroperitoneal paragangliomas from unicentric Castleman disease

**DOI:** 10.1038/s41598-020-69854-7

**Published:** 2020-07-30

**Authors:** Yuanyuan Jiang, Guozhu Hou, Zhaohui Zhu, Li Huo, Wuying Cheng, Fang Li

**Affiliations:** 1 Department of Nuclear Medicine, Peking Union Medical College Hospital Chinese Academy of Medical Sciences and Peking Union Medical College, Beijing, 100730 China; 2 Beijing Key Laboratory of Molecular Targeted Diagnosis and Therapy in Nuclear Medicine, Beijing, 100730 China

**Keywords:** Molecular medicine, Oncology

## Abstract

The aim of this study was to investigate the value of multiple parameters retrieved from the FDG PET/CT studies, including SUVmax, SUVmean, SUVpeak, MTV, TLG, tumour size in differentiating retroperitoneal paragangliomas from UCD. 28 patients with solitary retroperitoneal masses who had undergone preoperative ^18^F-FDG PET/CT were retrospectively evaluated. Histopathology by surgical resection confirmed 17 paragangliomas and 11 UCDs. SUVmax, SUVmean, SUVpeak, MTV, TLG, and tumour size of ^18^F-FDG PET/CT were measured for each patient. Mann–Whitney U-test was used to assess differences in multiple parameters between paragangliomas and UCDs. The ROC curve analysis was performed to determine the differential diagnostic value of these parameters. Paragangliomas presented significantly higher SUVmax (*P* < 0.001), SUVmean (*P* = 0.001), SUVpeak (*P* < 0.001), and TLG (*P* = 0.024) than UCDs, whereas no significant difference was observed in MTV. The AUCs for differentiating paragangliomas and UCDs were 0.920, 0.888, 0.909, and 0.765 for SUVmax, SUVmean, SUVpeak, and TLG, respectively. The SUVmax cut-off of 7.75 yielded 82.4% sensitivity and 100% specificity for predicting paragangliomas. This study indicated that ^18^F FDG PET/CT-derived multiple metabolic parameters are useful in distinguishing between paragangliomas and UCDs. SUVmax showed the best result for the differential diagnosis of these two diseases among multiple metabolic parameters.

## Introduction

Paragangliomas of the retroperitoneum are closely related to the inferior vena cava and aorta following the aorta-sympathetic chain^1^. They can be classified into functional and non-functional paragangliomas, and functional tumors are often associated with the symptoms of hypertension, tachycardia, headache, and diaphoresis^2^. However, non-functional paragangliomas can be completely clinically silent, and the levels of catecholamines have been found to be normal in these cases. They are also referred to as “incidentalomas”. Paragangliomas are highly vascular lesions and generally showed dramatic enhancement on contrast-enhanced Computed Tomography (CT) or Magnetic Resonance Imaging (MRI). Castleman disease (CD) is a rare lymphoproliferative disorder that was first described in 1956^3^. It is also known as angiofollicular or giant lymph node hyperplasia. CD is classified clinically as unicentric (UCD) or multicentric (MCD), and pathologically as hyaline vascular (HV) variant or plasma cell (PC) variant, or mixed variant^4,5^. Patients with UCD present with a solitary mass, and pathological examinations of UCD usually reveals HV features. UCD is most commonly found in the mediastinum (60 ± 70%)^5^. Abdominal forms are relatively rare (10 ± 17%) with the majority being retroperitoneal. Patients with UCD are usually asymptomatic and the disease is often an incidental finding at imaging. At CT, UCD often appears as a solitary soft-tissue mass with a well-defined border^6^. UCD is characteristically abundant with blood supply and tends to demonstrate intense enhancement during the arterial phase after administration of intravenous contrast material^6^. Therefore, it is difficult to differentiate retroperitoneal paragangliomas from UCDs using conventional cross-sectional imaging preoperatively due to the similar imaging features shared by these two entities^6–9^.

Paragangliomas could be visualized on somatostatin receptor imaging. However, UCDs have also been reported to be positive on somatostatin receptor imaging^7,10^, which makes the differentiation of these two entities difficult. The sensitivity of MIBG imaging in detecting paraganglioma was reported relatively low ranging from 56 to 72%^11,12^, resulting a relatively high false-negative rate. Negative findings of paragangliomas on MIBG are not uncommon. It is challenging to distinguish those paragangliomas presenting negative appearance on MIBG from UCDs. However, any physical contact with paragangliomas during the operation can induce cardiac arrhythmias and malignant hypertension^13^. Therefore, the differential diagnosis between them is crucial in decision making on a therapeutic strategy.

The description of fluorodeoxyglucose (FDG) positron emission tomography/ computed tomography (PET/CT) features of retroperitoneal paragangliomas and UCDs has been reported on case reports or small series in the literature, and no joint assessment has been performed before for the PET/CT appearances of these two diseases. Therefore, the aim of this retrospective study was to investigate whether ^18^F-FDG PET/CT-derived metabolic parameters played a role in differentiating retroperitoneal paragangliomas from UCDs. For this purpose, we compared the diagnostic value of metabolic parameters to identify the one with the highest diagnostic value in the differential diagnosis of these two diseases.

## Results

### Patient characteristics

**Figure 1:**
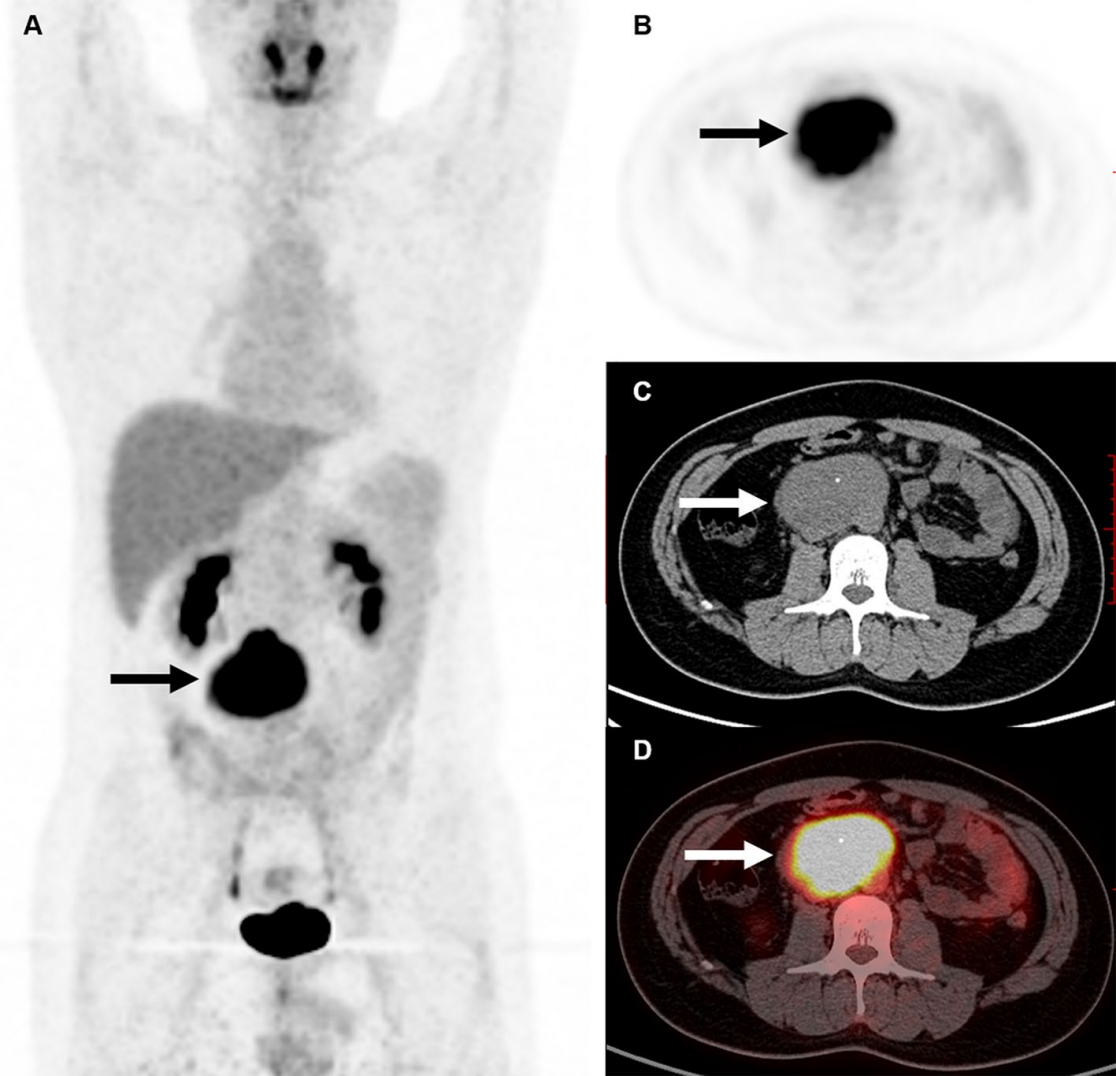
Representative case of a 32-year-old man with a retroperitoneal paraganglioma (patient 6). The patient underwent ^18^F-FDG PET/CT for characterizing the nature of the retroperitoneal solid mass incidentally detected by abdominal CT in a routine physical examination. The mass showed intense FDG activity with an SUVmax of 15.2 and a size of 8.1 cm (arrows).

**Figure 2:**
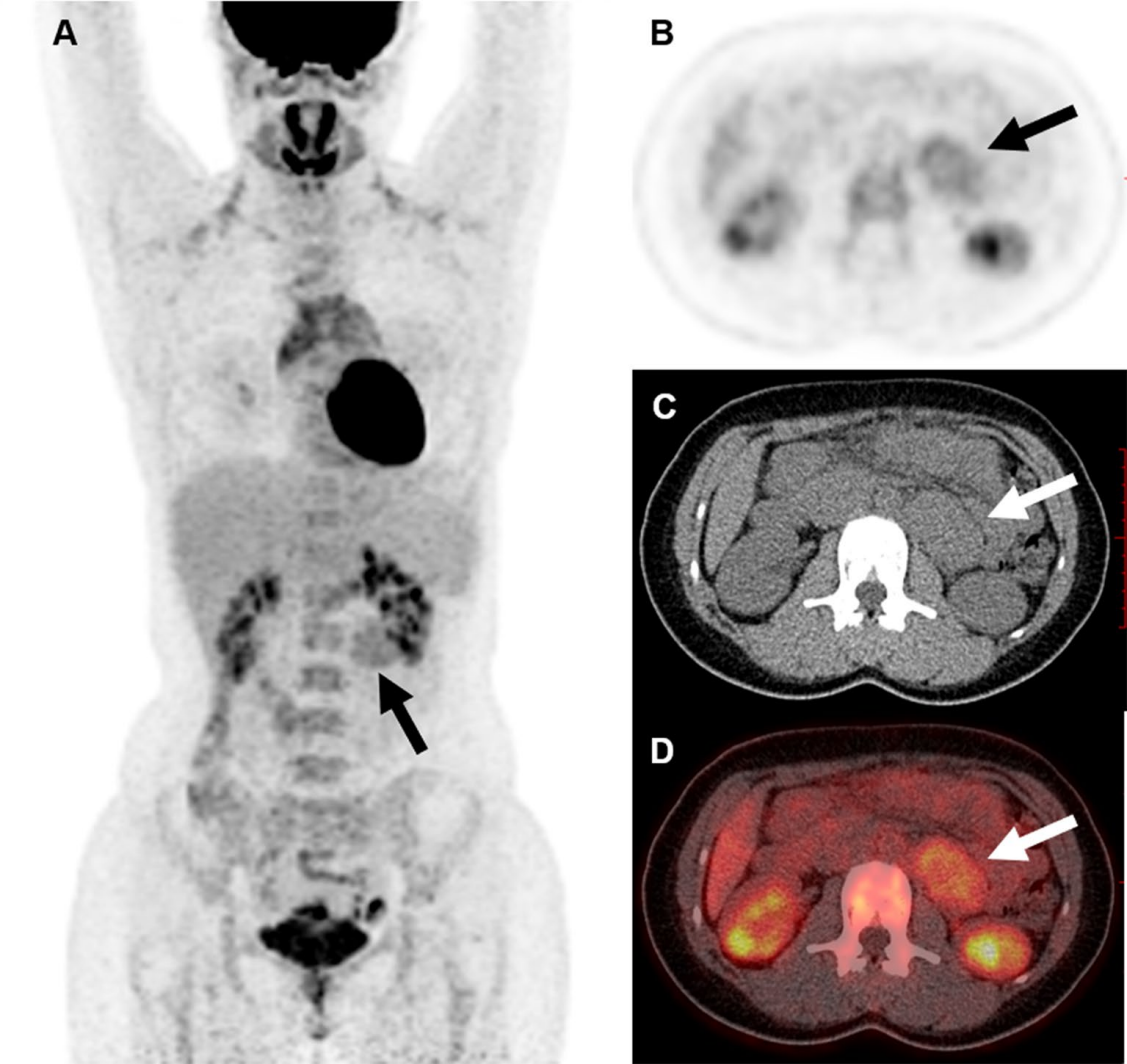
Representative case of a 25-year-old woman who underwent ^18^F-FDG PET/CT to evaluate a retroperitoneal solid mass (patient 27). The mass demonstrated moderately increased FDG uptake with an SUVmax of 3.2 and a size of 5.0 cm (arrows). Hypermetabolism was also seen in bilateral cervical and anterior mediastinal brown fat. The pathology confirmed the diagnosis of a Castleman’s disease, the hyaline vascular variant.

In total, 17 patients with retroperitoneal paraganglioma (Fig. 1) and 11 patients with UCD located in the retroperitoneum (Fig. 2) were included in the present study. Of the 17 patients with paraganglioma, 10 were female and 7 were male. Among the 11 patients with UCDs, 9 were female and 2 were male. The mean age of patients was 41.2 ± 11.3 (16‒56) years for the paraganglioma group and 39.6 ± 17.7 (12‒75) years for the UCD group (*P* = 0.771). 8 patients with UCDs were classified as hyaline vascular (HV) type, 1 as plasma cell (PC) type, and 2 as mixed variant. 10/17 of patients with paragangliomas underwent ^131^I-MIBG scan in this study. And ^131^I-MIBG scan showed positive findings in 8 of 10 patients. No patients with UCD underwent ^131^I-MIBG scan. Patient characteristics were shown in Table 1.

**Table 1:** Patient characteristics and PET/CT data.

Patient	Sex	Age (years)	Tumor size (cm)	SUVmax	SUVmean	SUVpeak	MTV	TLG	SUVmax of liver	SUVmax of mediastinal blood pool	Histopathology	Pathologic Subtypes	Ki-67 index	Genetic disorder	Clinical symptoms	Catecholamine secretion
1	M	56	4.7	12.7	6.6	7.8	10.5	69.6	3.5	2.6	Paraganglioma	–	20%	SDH-B	Symptomatic	Yes
2	F	47	3.7	35.2	23.4	22.4	13.2	308.8	2.7	1.8	Paraganglioma	–	< 1%	NA	Symptomatic	Yes
3	M	46	9.1	12.8	7.5	6.6	89.1	669	3	2.4	Paraganglioma	–	NA	NA	Symptomatic	Yes
4	F	16	6.5	24.5	17.5	20.4	43.4	757.5	2.4	2.1	Paraganglioma	–	5%	NA	Symptomatic	Yes
5	M	42	7.8	26.3	13.5	18.6	38.6	520.1	1.9	1.5	Paraganglioma	–	40%	NA	Symptomatic	Yes
6	M	32	8.1	15.2	8.5	7.5	96.9	823	2.8	1.9	Paraganglioma	–	2%	NA	Asymptomatic	No
7	F	53	6.0	22.6	12.4	17.1	55.6	691.6	2.7	1.8	Paraganglioma	–	< 1%	SDH-B	Symptomatic	Yes
8	M	34	7.2	37.6	25.1	36.2	59.4	1,488.4	3.1	2.1	Paraganglioma	–	< 1%	NA	Symptomatic	Yes
9	F	42	5.1	7.8	4	5.3	22.5	90.6	3.1	1.8	Paraganglioma	–	1%	NA	Symptomatic	Yes
10	F	49	7.5	13.6	8.9	6.5	95	843.1	2.6	1.8	Paraganglioma	–	1%	NA	Symptomatic	Yes
11	F	45	7.8	17	10.1	14.6	73.3	743	2.7	1.8	Paraganglioma	–	10%	NA	Symptomatic	Yes
12	F	51	2.8	4.6	2.8	3.7	16.2	44.7	2.6	1.9	Paraganglioma	–	1%	NA	Symptomatic	Yes
13	F	25	4.2	5.9	3.5	4.8	12.8	45	2.7	1.7	Paraganglioma	–	NA	NA	Symptomatic	Yes
14	F	50	13.2	13.4	8	11.5	99	790	2.8	2	Paraganglioma	–	2%	NA	Asymptomatic	No
15	M	47	4.5	4.3	2.6	3.5	29	76.3	2.9	1.6	Paraganglioma	–	< 1%	NA	Asymptomatic	No
16	F	24	4.8	18.3	11.7	16.1	36.4	425.8	2.3	1.7	Paraganglioma	–	10%	NA	Asymptomatic	No
17	M	42	2.3	12.5	7.6	8.7	4.6	35.1	2.4	1.6	Paraganglioma	–	< 1%	NA	Symptomatic	No
18	F	30	10.7	2.3	1.6	2.1	318.3	520.7	2.2	1.5	UCD	HV	10%	–		
19	F	50	9.2	7.7	3.8	4	161.4	619.8	3	2.1	UCD	Mixed	5%	–		
20	M	75	3.3	2.7	1.6	2	24.3	38.6	3.2	2.4	UCD	HV	10%	–		
21	M	34	2.2	7.5	4.9	5	7.6	37	3.5	2.6	UCD	HV	5%	–		
22	F	51	6.4	4	NA	NA	NA	NA	NA	NA	UCD	HV	40%	–		
23	F	58	4.2	4.9	2.8	3.4	32.2	89.9	4.3	3	UCD	HV	10%	–		
24	F	41	2.5	5.7	3.3	4.6	6.2	20.7	2.1	1.5	UCD	HV	NA	–		
25	F	31	6.2	6.1	4.5	5.8	46.2	208.4	2.1	1.7	UCD	PC	NA	–		
26	F	29	3.0	4	2.8	3.7	10.2	29	2.3	1.6	UCD	Mixed	80%	–		
27	F	25	5.0	3.2	2	1	47.2	94.9	2.3	1.3	UCD	HV	NA	–		
28	F	12	3.7	5.5	3.7	5.1	15.4	57.5	1.5	1	UCD	HV	NA	–		

*SUV* standardized uptake value, *MTV* metabolic tumour volume, *TLG* total lesion glycolysis, *UCD* unicentric Castleman disease, *NA* not available, *HV* hyaline vascular, *PC* plasma cell, *SDH* succinate dehydrogenase. Symptomatic meant that patients have catecholamine-secreting associated symptoms, including hypertension, dizziness, palpitation, or perspiration, etc.

### PET/CT performance

**Figure 3:**
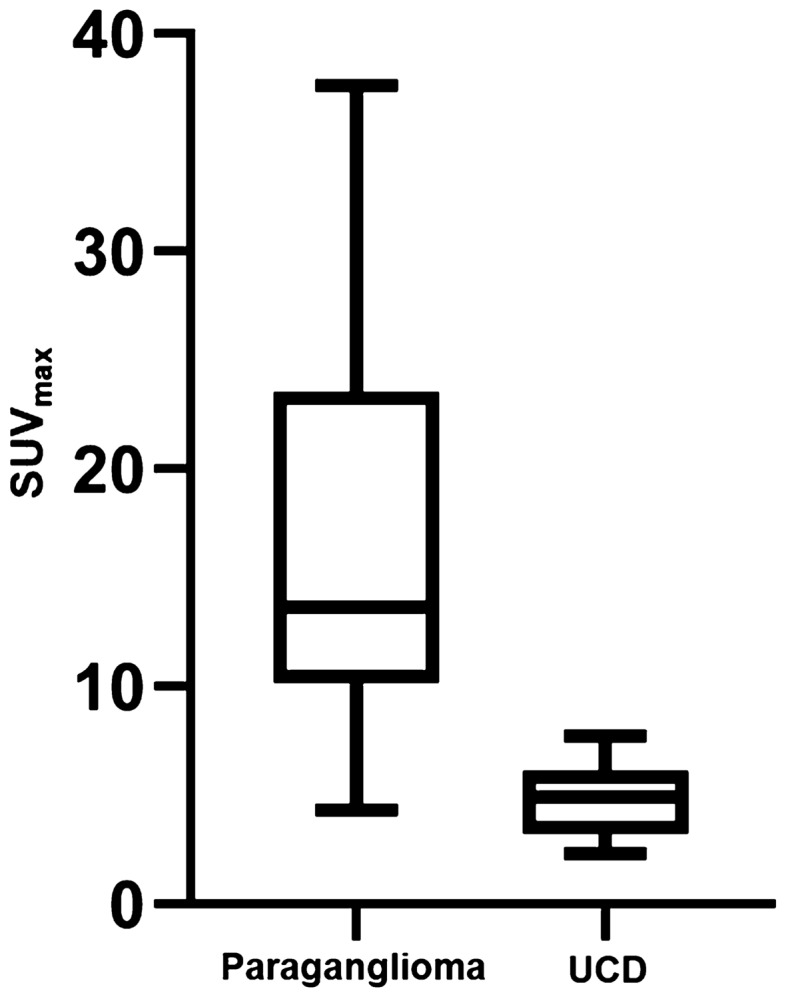
SUVmax was significantly higher in paragangliomas than in unicentric Castleman disease (UCD) (*P* < 0.001).

Varying degrees of FDG activity was demonstrated in all paragangliomas and UCDs. The comparison of characteristics of paragangliomas and UCDs were summarized in Table 2. For the 17 patients with paragangliomas, the average SUVmax was 16.7 ± 9.8 ranging from 4.3 to 37.6. For the 11 patients with UCDs, the average SUVmax was 4.9 ± 1.8 ranging from 2.3 to 7.7. The SUVmax was significantly higher in paragangliomas compared with that in UCDs (*P *< 0.001; Fig. 3). SUVmean [10.2 ± 6.6 (range: 2.6–25.1) vs 3.1 ± 1.2 (range: 1.6–4.9), *P* = 0.001], SUVpeak [12.4 ± 8.7 (range: 3.5–36.2) vs 3.7 ± 1.6 (range: 1.0–5.8), *P* < 0.001], and TLG[495.4 ± 409.2 (range: 35.1–1,488.4) vs 171.7 ± 218.2 (range: 20.7–619.8), *P* = 0.024] were also significantly higher in paragangliomas than in UCDs.

**Table 2:** Comparison of characteristics of paragangliomas and UCDs.

	Paragangliomas (n = 17)	UCDs (n = 11)	*P* value
Age (years)	41.2 ± 11.3	39.6 ± 17.7	0.771
Gender (M:F)	7:10	2:9	0.211
SUVmax	16.7 ± 9.8	4.9 ± 1.8	< 0.001
SUVmean	10.2 ± 6.6	3.1 ± 1.2	0.001
SUVpeak	12.4 ± 8.7	3.7 ± 1.6	< 0.001
MTV	46.8 ± 33.3	66.9 ± 99.5	0.581
TLG	495.4 ± 409.2	171.7 ± 218.2	0.024
Tumor size (cm)	6.2 ± 2.7	5.1 ± 2.8	0.318
Ki-67 index (%)	6.5 ± 10.7	22.9 ± 27.9	0.057

*UCD* unicentric Castleman disease, *SUVmax* maximum standardized uptake value.

There was no significant difference (*P* = 0.581) in MTV between paragangliomas (46.8 ± 33.3, range: 4.6–99) and UCDs (66.9 ± 99.5, range: 6.2–318.3). The lesion size was 6.2 ± 2.7 cm (range: 2.3–13.2 cm) for paragangliomas and 5.1 ± 2.8 (range: 2.2–10.7 cm) for UCDs (*P* = 0.318). There was also no statistically significant difference in the FDG uptake between the 4 non-functioning paragangliomas and the remaining 13 functioning ones (12.8 ± 6.0 vs 17.9 ± 10.6; *P* = 0.377).

According to the histological results, Ki-67 proliferation indices were available for 22 patients (15 paragangliomas, 7 UCDs). The Ki-67 index was higher in UCDs than paragangliomas (6.5 ± 10.7% vs 22.9 ± 27.9%), while the difference was not statistically significant (*P* = 0.057). In addition, no significant correlation between Ki-67 index and all 5 metabolic parameters were revealed neither in the paraganglioma group (R^2^ range: 0.097–0,250, *P* > 0.05) nor in the UCD group (R^2^ range: – 0.636 to – 0.031, *P* > 0.05).

### ROC analysis of metabolic parameters

**Figure 4:**
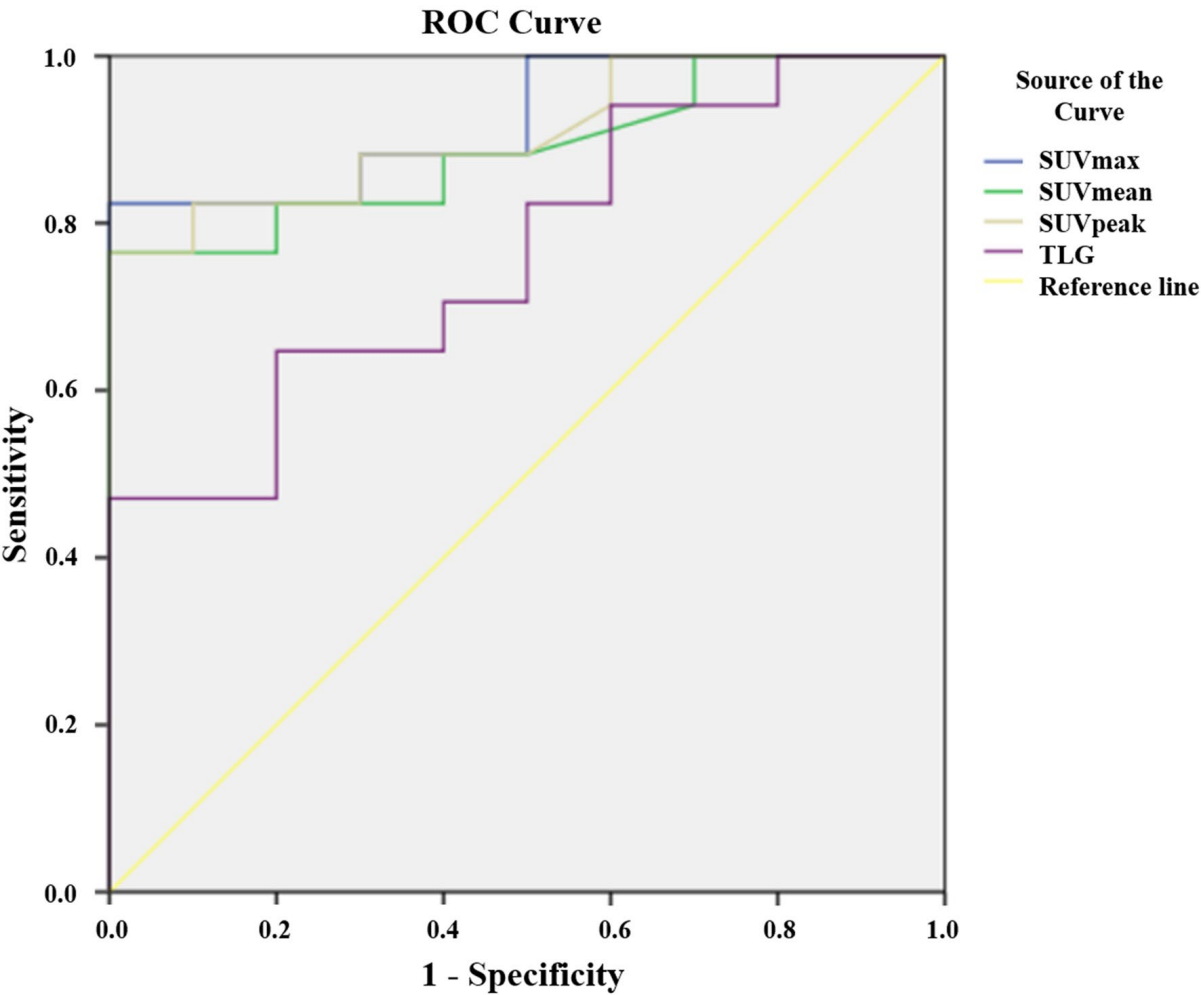
Receiver operating characteristic curves for measuring the accuracy of the SUVmax, SUVmean, SUVpeak, and TLG as parameters for distinguishing paraganglioma from unicentric Castleman disease (UCD). The area under the curve is 0.920, 0.888, 0.909, and 0.765 for SUVmax, SUVmean, SUVpeak, and TLG, with a cut-off point of 7.75, 5.75, 76.5, and 258.6, respectively.

Table 3 summarizes the sensitivity, specificity of SUVmax, SUVmean, SUVpeak, and TLG. The ROC curve analysis showed that the SUVmax had the best diagnostic performance for predicting paragangliomas on the basis of the AUC. To differentiate paragangliomas from UCDs, SUVmax displayed the highest AUC value of 0.920 (*P* < 0.001; 95%CI: 0.819–1.000) among metabolic parameters with a sensitivity of 82.4% and a specificity of 100% at a cut-off of 7.75 (Fig. 4). The AUC value for SUVmean was 0.888 (*P* = 0.001; 95%CI: 0.765–1.000) at a cut-off of 5.75 with 76.5% sensitivity and 100% specificity. SUVpeak (AUC: 0.909; *P* < 0.001; 95% CI: 0.800–1.000) had a sensitivity of 76.5, a specificity of 100% at a cut-off of 6.15. TLG had the lowest AUC value of 0.765 (*P* = 0.024) with 64.7% sensitivity and 80% specificity at a cut-off of 258.6.

**Table 3:** ROC curve analysis of metabolic parameters in differentiating paragangliomas and UCDs.

Metabolic parameters	Cut-off value	AUC	Sensitivity (%)	Specificity (%)	*P* value
SUVmax	7.75	0.920	82.4	100	< 0.001
SUVmean	5.75	0.888	76.5	100	0.001
SUVpeak	6.15	0.909	76.5	100	< 0.001
TLG	258.6	0.765	64.7	80	0.024

*ROC* receiver operating characteristic, *UCD* unicentric Castleman disease, *SUV* standardized uptake value, *TLG* total lesion glycolysis, *AUC* area under the receiver operating characteristic curve.

## Discussion

FDG PET findings on retroperitoneal UCDs have only been described in a few case reports or small series so far. These reports supported our results, showing that UCDs demonstrated moderate hypermetabolic activity with reported SUVmax ranging from 2.93 to 4.8^14–18^. A study by Lee et al. investigated metabolic characteristics of 12 patients with CDs (8 MCDs, 4 UCDs) and drew a conclusion similar to ours that CDs, especially UCDs, shows moderately increased activity on FDG PET/CT^19^. A few studies and case reports of FDG PETCT concerning retroperitoneal paragangliomas provided their metabolic information, revealing intense FDG activity to the tumor. Their findings were also in accordance with ours^20–26^. In this study, we tried to investigate the value of multiparameter FDG PET/CT imaging in differentiating paragangliomas and UCDs, which has not been reported in the current literature.

In the present study, 4 metabolic parameters (SUVmax, SUVmean, SUVpeak, TLG) were significantly higher than in paragangliomas than UCDs. Among these parameters, SUVmax showed the highest diagnostic value in the differential diagnosis between these two diseases. We found that a cut-off SUVmax of 7.75 maximized the sensitivity and specificity for differentiating paragangliomas from UCDs. Specifically, our findings indicate that a single retroperitoneal mass with an SUVmax higher than 7.75 is more likely to be a paraganglioma.

The question arises as to the phenomenon that underlies the difference in FDG uptake. Ki-67 was well known to be associated with the proliferative ability of the tumor. One speculation was a difference in the Ki-67 index since a high FDG uptake was reported to be correlated with a more proliferative index in some tumors^27–33^. However, we observed an interesting finding in the present study that the Ki-67 index was higher in UCDs (although statistically insignificant) than paraganglioma. No correlation between Ki-67 index and SUVmax value was observed neither in the UCD group nor in the paraganglioma group. In some UCD cases, the Ki-67 index was very high while the metabolic activity still remained moderate (patient 22, 26). On the other hand, in the paraganglioma group, we noticed that quite a few tumors exhibited very intense FDG activity on PET/CT while the Ki-67 proved to be as low as 1% or even less than 1%. This finding was in line with a previous study by Lin et.al, who reported that SUVmax value of pheochromocytoma and paraganglioma was not correlated with Ki-67^21^. These findings made Ki-67 theory an unlikely one. Further investigation was still required to figure out the reason why the UCDs demonstrated moderately increased FDG activity even with a considerably high Ki-67 index. A comparison between lesion size of these two diseases was also performed to see if it might exert an effect on the difference of FDG uptake. However, the comparison did not yield a statistically significant difference with the average size of paragangliomas only slightly larger than UCDs, making lesion size theory another unlikely one.

UCD often manifested as a solitary mass. Patients with UCD were generally asymptomatic or had only local symptoms associated with compression of adjacent structures and organs. Complete surgical resection of the tumor was curative and was associated with > 90% relapse-free survival^34^. Boutboul et al. suggested that in UCD patients for whom surgery is impossible, the “watch-and-wait” approach should be considered. They found that most of patients in whom such strategy was adopted remained stable, including one patient with a single stable mediastinal mass for 17 years^35^. Gonzalez-Garcia et al. retrospectively evaluated clinical and pathological characteristics of 53 patients with CD (20 UCD, 33MCD). They found that there were significantly lower relapses and mortality in UCD than in MCD, concluding that UCD represents a benign disorder^36^. Although under what mechanism that UCD showed such an FDG avidity remained unclear, our results seemed to indicate that PET/CT findings of UCDs corresponded to the nonprogressive and benign nature of the disease.

The majority of the paragangliomas in this study demonstrated intense FDG activity. The pathological molecular mechanism of FDG uptake in paraganglioma has not yet been clarified. Data from the related literature supports two explanations concerning the intense FDG avidity of paragangliomas, whether benign or malignant: pseudohypoxia model related to specific genetic defects (VHL, SDHx), as in patients 1 and 7 in our series, and adaptive responses to hypoxia in sporadic patients^37^. Some paragangliomas are caused by a germline mutation in SDH (SDHB, SDHC, and SDHD) or VHL tumor suppressor genes^38^. Mutations in these genes are associated with the induction of a hypoxic response under normal oxygen conditions, a response mediated by the oxygen-regulated transcription factor hypoxia-inducible factor 1a^39^. SDH and VHL mutations could result in the stabilization of hypoxia-inducible factor 1a leading to inhibition of the tricarboxylic acid cycle and increased glycolysis, which could in turn cause increased glucose demand and high FDG uptake (Warburg effect)^40^. Another explanation for such an SUVmax in paragangliomas might be that the preferential use of glycolysis by tumor cells may provide cells with competitive advantages under conditions of hypoxia^37,41^. The fact that these tumors are highly vascularized-a hallmark of a hypoxic response, was also in support of this theory^37^. Kaida et al. presented a case of sporadic paraganglioma in the retroperitoneum demonstrating intense FDG activity with SUVmax 30.5^26^. They observed that the red blood cells involved in the intra-tumor hemorrhage had a high expression of GLUT-1 and there were some macrophages with CD 68 expression in the tumor nest, concluding that FDG uptake in paraganglioma cells might be related to intra-tumor hemorrhage and macrophages^26^.

The primary limitations of the present study were the relatively small sample size, which does not allow for powerful statistical analysis, and retrospective design. Due to the study’s retrospective nature, the genetic disorder data of patients with paragangliomas was inadequate. Therefore, the mechanism of intense FDG activity for some paragangliomas remained unclear.

Based on the findings of this study, we conclude that FDG PET/CT-derived metabolic parameters (SUVmax, SUVmean, SUVpeak, TLG) were helpful in differentiating retroperitoneal paragangliomas from UCDs. Among these metabolic parameters, SUVmax showed the best result with rather high sensitivity and specificity. Still, a study of a larger sample size is needed to validate our results.

## Methods

### Ethics approval and consent to participate

This study was approved by the Institutional Review Board of Peking Union Medical College Hospital. The requirement for informed consent was waived due to its retrospective nature and it was confirmed by IRB of our institution. Investigations were carried out as per the rules of the Declaration of Helsinki of 1975, revised in 2013. All methods were performed in accordance with the institutional guidelines and regulations.

### Patients

A total of 28 patients with solitary retroperitoneal masses who had undergone preoperative ^18^F-FDG PET/CT between May 2012 and October 2019 in our department were retrospectively evaluated. Histopathology by surgical resection confirmed 17 paragangliomas and 11 UCDs. The pathologic subtype and Ki-67 index of UCD was determined by a review of pathologic reports.

### ^18^F-FDG PET/CT Study

Following 8 h of fasting with blood glucose level of less than 120 mg/dL, all patients were intravenously injected with ^18^F-FDG (5.5 MBq/kg). The patients then rested in a quiet room for nearly 1 h. Imaging was performed using a Siemens Biograph 64 Truepoint PET/CT scanner. First tomogram images, then a low-dose CT scan and finally PET images from the mid-thigh to skull base were acquired. CT scan was obtained for attenuation correction with a tube voltage of 120 kV, a tube current of 80 mA, and scanning thickness of 3 mm. The PET acquisition was acquired in 3-dimensional mode, 2 min per bed position. The raw data were reconstructed using an ordered-subset expectation–maximization (OSEM) iterative reconstruction algorithm.

### Image interpretation and statistical analysis

All the PET/CT images were reviewed by two experienced nuclear medicine physicians. A region of interest (ROI) was drawn around the retroperitoneal lesion while avoiding the peripheral area. All values of maximum standardized uptake value (SUVmax), mean standardized uptake value (SUVmean), peak standardized uptake value (SUVpeak), metabolic tumour volume (MTV), total lesion glycolysis (TLG) were measured by the analysis software (Medex-NM imaging analysis system) for each lesion. The size of each lesion was determined as the longest diameter based on CT scans. All data were expressed as mean ± SD. Statistical differences between groups were investigated using a Mann–Whitney U-test. The association between Ki-67 and metabolic parameters was analyzed through Spearman correlation. The cut-off value of metabolic parameters for differentiating retroperitoneal paraganglioma from UCD was obtained via the receiver operating characteristic (ROC) analysis. The areas under the curve and the sensitivity and specificity of differential diagnoses were also calculated. For all tests, *P *value < 0.05 was considered statistically significant. All statistical analyses were performed using SPSS (IBM SPSS Statistics for Windows, Version 21.0. Armonk, NY).

## Data Availability

The data that support the findings of this study are available from the corresponding author, upon reasonable request.
