# Author Correction: The value of multiparameter ^18^F-FDG PET/CT imaging in differentiating retroperitoneal paragangliomas from unicentric Castleman disease

**DOI:** 10.1038/s41598-021-84453-w

**Published:** 2021-02-23

**Authors:** Yuanyuan Jiang, Guozhu Hou, Zhaohui Zhu, Li Huo, Wuying Cheng, Fang Li

**Affiliations:** 1 Department of Nuclear Medicine, Peking Union Medical College Hospital Chinese Academy of Medical Sciences and Peking Union Medical College, Beijing, 100730 China; 2 Beijing Key Laboratory of Molecular Targeted Diagnosis and Therapy in Nuclear Medicine, Beijing, 100730 China

Correction to: *Scientific Reports* 10.1038/s41598-020-69854-7, published online 30 July 2020

The original version of this Article contained an error in the order of corresponding authors, which was incorrectly given as Fang Li & Wuying Cheng. This error has now been corrected in the PDF and HTML versions of the Article.

